# Reduction of Dark Current in CMOS Image Sensor Pixels Using Hydrocarbon-Molecular-Ion-Implanted Double Epitaxial Si Wafers

**DOI:** 10.3390/s20226620

**Published:** 2020-11-19

**Authors:** Ayumi Onaka-Masada, Takeshi Kadono, Ryosuke Okuyama, Ryo Hirose, Koji Kobayashi, Akihiro Suzuki, Yoshihiro Koga, Kazunari Kurita

**Affiliations:** SUMCO Corporation, 1-52 Kubara, Yamashiro-cho, Imari-shi, Saga 849-4256, Japan; tkadono@sumcosi.com (T.K.); rokuyama@sumcosi.com (R.O.); rhirose@sumcosi.com (R.H.); kkobayas@sumcosi.com (K.K.); asuzuki1@sumcosi.com (A.S.); ykoga4@sumcosi.com (Y.K.); k-kurita@sumcosi.com (K.K.)

**Keywords:** CMOS image sensor, gettering, white spot defects, oxygen

## Abstract

The impact of hydrocarbon-molecular (C_3_H_6_)-ion implantation in an epitaxial layer, which has low oxygen concentration, on the dark characteristics of complementary metal-oxide-semiconductor (CMOS) image sensor pixels was investigated by dark current spectroscopy. It was demonstrated that white spot defects of CMOS image sensor pixels when using a double epitaxial silicon wafer with C_3_H_6_-ion implanted in the first epitaxial layer were 40% lower than that when using an epitaxial silicon wafer with C_3_H_6_-ion implanted in the Czochralski-grown silicon substrate. This considerable reduction in white spot defects on the C_3_H_6_-ion-implanted double epitaxial silicon wafer may be due to the high gettering capability for metallic contamination during the device fabrication process and the suppression effects of oxygen diffusion into the device active layer. In addition, the defects with low internal oxygen concentration were observed in the C_3_H_6_-ion-implanted region of the double epitaxial silicon wafer after the device fabrication process. We found that the formation of defects with low internal oxygen concentration is a phenomenon specific to the C_3_H_6_-ion-implanted double epitaxial wafer. This finding suggests that the oxygen concentration in the defects being low is a factor in the high gettering capability for metallic impurities, and those defects are considered to directly contribute to the reduction in white spot defects in CMOS image sensor pixels.

## 1. Introduction

Complementary metal-oxide-semiconductor (CMOS) image sensors are widely used in various applications such as in vehicles, medical equipment, and consumer cameras. There is a high demand for the fabrication of high-performance CMOS image sensors with characteristics such as high sensitivity, high resolution, and high-speed image data processing owing to the expansion of the CMOS image sensor market. Recently, three-dimensional (3D)-stacked backside illuminated (BSI) CMOS image sensors allowing multiple functions of image sensors have attracted attention [1,2,3]. The fabrication of 3D-stacked CMOS image sensors is achieved by 3D integration technology that enables the stacking of different kinds of circuit blocks, such as sensor, memory, and logic blocks, into one chip with mainly Cu through-silicon via (TSV) technology. However, the using of Cu TSV technology may cause Cu contamination during the TSV fabrication process and when the property of the barrier layer is insuffcient for preventing Cu diffusion [4,5]. In addition, during the fabrication of BSI-based 3D stacked CMOS image sensors, the device Si wafer must be thinned by mechanical grinding and chemical mechanical polishing (CMP). The thin-wafer fabrication process has a risk of degradation of the electrical performance of the CMOS image sensor owing to easy metallic impurity contamination into the active region of the photodiode [6,7,8]. Therefore, the fabrication process of 3D-stacked BSI image sensors has many chances for metallic impurities to be introduced compared with that of conventional front-side illuminated (FSI) image sensors. It is well known that metallic impurities form deep-energy-level defects in the Si band gap. These defects allow the thermal generation of carriers, resulting in dark current, which is a key electrical parameter in determing the performance of CMOS image sensors [9,10,11,12,13]. The large dark current generated from the photodiode is known to cause blemished pixels called white spot defects. Therefore, a Si wafer with much a higher gettering capability is required to for suppressing metallic-impurity-related defect formation in the active region of devices and fabricate the high-performance 3D-stacked BSI image sensors.

Our research group has been developing novel gettering Si wafers by a hydrocarbon-molecular-ion implantation technique using C_3_H_5_ or C_3_H_6_ ions to improve the electrical performance of CMOS image sensors [14,15,16]. The novel gettering Si wafers comprise an epitaxial Si wafer implanted with hydrocarbon molecular ions in the Czochralski (CZ)-Si substrate. It was found that such implantation results in the formation of effective gettering sinks for metallic impurities, such as Fe, Cu, Ni, oxygen (O), and hydrogen (H), under the active region of CMOS image sensors. For a high gettering capability for metallic impurities, the hydrocarbon-molecular-ion-implanted Si wafer can have reduced white spot defects compared with an intrinsic gettering (IG) silicon wafer, which is currently the most common gettering technique in the semiconductor industry. The gettering sinks formed by hydrocarbon-molecular-ion implantation can also act as a diffusion barrier preventing O diffusion from the Czochralski (CZ)-Si substrate by the effective gettering of oxygen impurities. This leads to the reduction in white spot defects [17,18,19]. Additionally, H atoms gettered in the hydrocarbon-molecular-ion-implanted region can reduce the density of interface state defects at the Si/SiO_2_ interface by passivating the defects during heat treatment [20,21]. This leads to the reduction in dark current and random telegraph signal (RTS) noise.

Additionally, we have recently demonstrated that hydrocarbon-molecular-ion implantation in an epitaxial growth layer with a low O concentration, rather than in the CZ-Si substrate, resulted in a higher gettering capability for intentionally contaminated Fe [22]. It was found that the gettering capability for Fe is determined by the O concentration in the hydrocarbon-molecular-ion implantation region. The implantation in the epitaxial growth layer results in a double epitaxial Si wafer, with a hydrocarbon-molecular-ion-implanted first epitaxial layer on which a second epitaxial layer is grown. It is expected that the hydrocarbon-molecular-ion-implanted Si wafer with a double-epitaxial-layer structure, which has a higher gettering capability for metallic impurities, can solve the technical issue caused by metallic-impurity-related defect formation in 3D-stacked BSI CMOS image sensors. However, it is unclear whether the hydrocarbon-molecular-ion-implanted double epitaxial Si wafer can contribute to improving the performance of CMOS image sensor pixels. The electrical performances of CMOS image sensor pixels, such as the white spot defects and dark current of the hydrocarbon-molecular-ion-implanted double epitaxial Si wafer, must be investigated because we have not yet obtained direct evidence about the metal-gettering behavior in the device fabrication process.

Dark current spectroscopy (DCS) is well known to be an extremely powerful method for analyzing the metallic impurity contamination in charge-coupled devices (CCDs) and CMOS image sensors [23,24,25,26,27,28]. The dark current intensity in image sensors evaluated by DCS strongly depends on the density of metallic-impurity-related defects in the space charge region of the photodiode. The detection of dark current caused by impurity-related defects in the Si band gap by DCS was first proposed by McGrath et al. [23]. It is based on the assumption that the amount of dark current generated at a certain temperature is a value specific to the impurity-related defects. In this study, therefore, we used white spot defects and dark current measured by DCS as a parameter to investigate the impact of O concentration in the hydrocarbon-molecular-ion-implanted region on the electrical performance of CMOS image sensor pixels. The white spot defects and dark current of the hydrocarbon-molecular-ion-implanted double epitaxial Si wafer were compared with those of a conventional single epitaxial Si wafer and an epitaxial Si wafer without implantation. Moreover, we investigated the characteristics of O and H gettering on the hydrocarbon-molecular-ion-implanted double epitaxial Si wafer, the barrier effect of O diffusion by O gettering, and the concentration of H diffusion from the implanted region, which contribute to the reduction in interface state defects. Finally, in this paper, the appropriate design of the Si wafer gettering layer for solving the 3D-stacked BSI CMOS image sensor technical issue is proposed.

## 2. Materials and Methods

**Figure 1:**
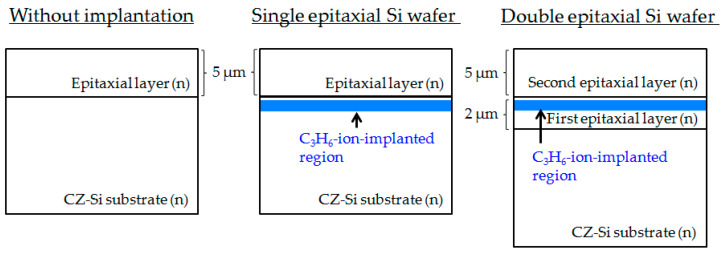
Cross-sectional schematic illustrations of epitaxial wafers used in this study.

The experimental silicon wafers used in this study are schematically shown in Figure 1. We used phosphorus-doped epitaxial Si wafers with epitaxial layers 5 μm thick for all epitaxial Si wafers. Single epitaxial Si wafers were fabricated with hydrocarbon-molecular-ion implantation in the phosphorus and carbon (C)-doped CZ-Si substrate. Double epitaxial Si wafers were fabricated with hydrocarbon-molecular-ion implantation in the first phosphorus-doped epitaxial growth layer of 2 μm thickness, and then the second epitaxial layer was deposited on the first epitaxial layer. The CZ-Si substrate fabricated with the double epitaxial Si wafer was the same as those of the single epitaxial Si wafer and the epitaxial wafer without implantation. The concentrations of oxygen (O) and C in the CZ-Si substrate were 1.4 × 10^18^ and 3 × 10^16^ cm^−3^, respectively. Hydrocarbon molecular ions were implanted into the wafer to a C dose of 1 × 10^15^ cm^−2^ with an energy of 80 keV using an ion implanter (Nissin Ion Equipment CLARIS) [29,30]. The C_3_H_6_ ion was selected as the hydrocarbon molecular ion in this study because C_3_H_6_ ions can implant with higher beam current than other hydrocarbon molecular ions. The C_3_H_6_ beam current was 1700 μA.

DCS measurements were performed at 60 °C using the 4T-type pinned photodiode CMOS image sensors with 3 μm pixel size fabricated by the CMOS image sensor fabrication process. CMOS image sensors were fabricated by a 65 nm process node. White spot defects and dark currents of image sensors were evaluated by DCS. A white spot defect was defined as the cumulative number of pixels showing dark current generation levels exceeding 35 electrons/s in the dark current distribution measured by DCS. Dark current was defined as the median of DCS.

The density of O precipitate defects, called bulk microdefects (BMDs), in the CZ-Si substrate and the concentration depth profiles of the metallic impurities C, O, and H were measured on samples that underwent DCS measurements. The density of BMD in the CZ-Si substrate was measured by optical microscopy observation (Raytex Optima MO441, Woodbury, NY, USA). The concentration depth profiles in the C_3_H_6_-ion-implanted region were obtained by secondary ion mass spectrometry (SIMS) analysis, and the surface metal layer of samples was mechanically polished to a depth of about 0.5 μm before analysis. The concentration depth profiles of O and H before device fabrication were also obtained by SIMS analysis.

**Figure 2:**
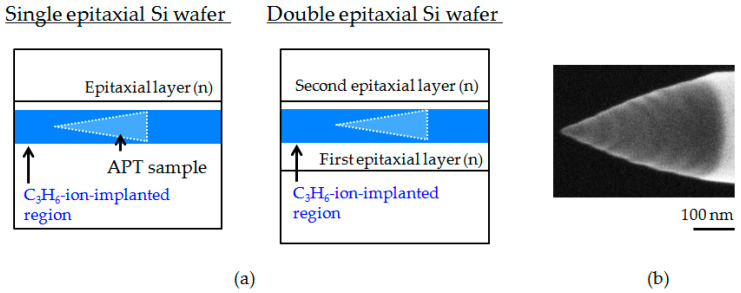
(**a**) Schematic illustration of laser-assisted atom probe tomography (L-ATP) analysis region for C_3_H_6_-ion-implanted single and double epitaxial Si wafers. (**b**) Acicular sample for L-APT analysis.

The structure of C_3_H_6_-ion-implantation-related defects was analyzed by high-resolution transmission electron microscopy (TEM) (Hitachi H-9000UHR-I, Tokyo, Japan). The morphology of C_3_H_6_-ion-implantation-related defects at the atomic level was analyzed by laser-assisted atom probe tomography (L-APT) (AMETEC LEAP 4000XSi, Berwyn, PA, USA) with an ultraviolet laser (wavelength: 355 nm). Acicular L-APT samples were lifted out from the C_3_H_6_-ion-implanted region located approximately 5 μm from the sample surface, using a focused ion beam system, as shown in Figure 2. The APT data were analyzed using integrated visualization and analysis software (IVAS) from CAMECA.

The C_3_H_6_-ion implantation-related electronic defects in single and double epitaxial Si wafers before device fabrication were investigated by measuring room temperature photoluminescence (RTPL) (WaferMasters MPL300, San Jose, CA, USA) using 670 and 827 nm excitation with penetration depths of ~4 and ~10 μm from the wafer surface, respectively. The RTPL spectra were measured in the wavelength range of 900–1400 nm.

## 3. Results and Discussion

### 3.1. Gettering Capability of C_3_H_6_-Ion-Implanted Double Epitaxial Si Wafers

**Figure 3:**
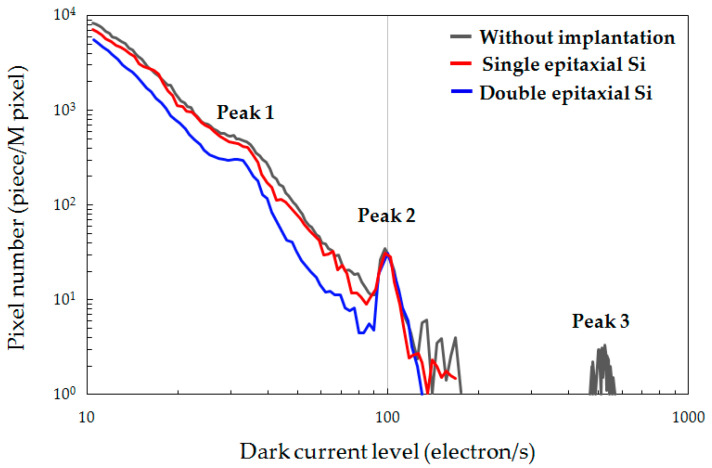
Dark current distribution measured by DCS at 60 °C for complementary metal-oxide-semiconductor (CMOS) image sensors of epitaxial Si wafer without implantation and C_3_H_6_-ion-implanted single and double epitaxial Si wafers.

Figure 3 shows DCS spectra at 60 °C of the epitaxial Si wafer without implantation and C_3_H_6_-ion-implanted single and double epitaxial Si wafers. The epitaxial Si wafer without implantation used in this study is the IG-enhanced epitaxial Si wafer that has BMD in the Si bulk. The white spot defect number of the C_3_H_6_-ion-implanted double epitaxial Si wafer was the lowest at any dark signal level except 100 electrons/s. Additionally, the DCS spectra exhibited three peaks at dark signal levels of approximately 35, 100, and 530 electrons/s, which we labeled 1, 2, and 3, respectively. The white spot defect numbers of Peak 1 and 3 of both the C_3_H_6_-ion-implanted single and double epitaxial Si wafers were lower than that of the epitaxial Si wafer without implantation. In particular, Peak 1 was the lowest in the C_3_H_6_-ion-implanted double epitaxial Si wafer. On the other hand, the white spot defect number of Peak 2 did not indicate any difference among all gettering techniques. It is considered that Peak 2 corresponds to the dark current induced by process-induced defects and not metallic-impurity-related defects.

**Figure 4:**
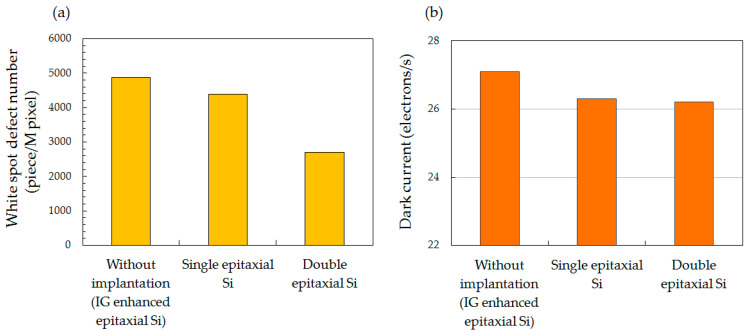
Comparison of (**a**) white spot defect number exceeding 35 electrons/s and (**b**) dark current for CMOS image sensors of epitaxial Si wafer without implantation and C_3_H_6_-ion-implanted single and double epitaxial Si wafers.

To more easily compare DCS characteristics, bar graphs of white spot defects exceeding 35 electrons/s and dark current in C_3_H_6_-ion-implanted single and double epitaxial Si wafers are shown in Figure 4. That of the epitaxial Si wafer without C_3_H_6_-ion implantation is also shown in Figure 4 for comparison. The white spot defects shown in Figure 4a of both the C_3_H_6_-ion-implanted single and double epitaxial Si wafers were lower than that of the epitaxial Si wafer without implantation. The white spot defects of the single epitaxial Si wafer, compared to that of the IG-enhanced epitaxial Si wafer, was in good agreement with a previous study in which dark current was evaluated to exceed 100 electrons/s [16]. However, the reduction in white spot defects of the double epitaxial Si wafer was the largest among gettering techniques. The white spot defect of the double epitaxial Si wafer was 40% lower than that of the single epitaxial Si wafer. Additionally, although the dark currents shown in Figure 4b of both the C_3_H_6_-ion-implanted single and double epitaxial Si wafers were lower than that of the epitaxial Si wafer without implantation, there was no large difference between single and double epitaxial Si wafers.

It is considered that generation of white spot defects is due to carrier generation via the impurity-related deep levels in the space charge region of the photodiode [23,27,28]. This means that fewer white spot defects indicate a lower concentration of impurity-related defects in the active region of devices. Therefore, it is considered that the C_3_H_6_-ion-implanted double epitaxial Si wafer has the most suppressed impurity-related defect formation in the active region of devices compared with the C_3_H_6_-ion-implanted single epitaxial Si wafer and the epitaxial Si wafer without implantation, probably because of the difference in the ease at which gettering sinks formed in each type of Si wafer.

The C_3_H_6_-ion-implanted double epitaxial Si wafer is expected to have two regions of possible gettering sinks for metallic impurities, i.e., C_3_H_6_-ion-implanted regions and the BMD formation region. It is considered that the difference in the gettering capabilities of the two C_3_H_6_-ion-implanted regions and the BMD formation region causes the difference in white spot defect characteristics of CMOS image sensor pixels.

**Figure 5:**
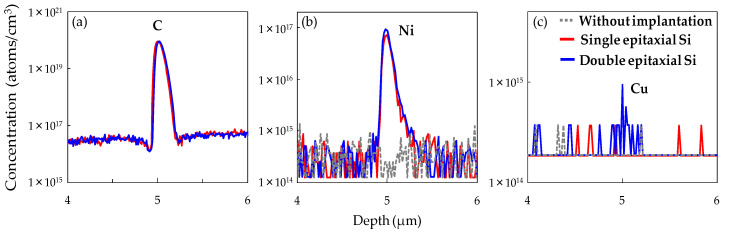
SIMS depth profiles of (**a**) C concentration, (**b**) Ni concentration, and (**c**) Cu concentration in C_3_H_6_-ion-implanted regions of single and double epitaxial Si wafers after device fabrication.

**Figure 6:**
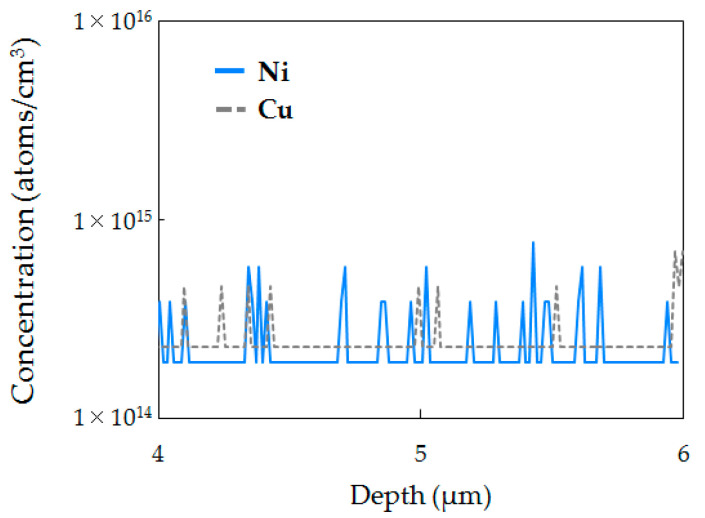
SIMS depth profiles of Ni and Cu concentrations of epitaxial wafers before device fabrication.

First, the gettering behavior for metallic impurities on C_3_H_6_-ion-implanted regions is investigated. Figure 5a–c respectively shows the SIMS depth profiles of C, Ni, and Cu concentrations in the epitaxial Si wafer without implantation (IG enhanced epitaxial Si wafer) and C_3_H_6_-ion-implanted single and double epitaxial Si wafers after the device fabrication processes. SIMS profiles show that Ni and Cu were gettered in the C_3_H_6_-ion-implanted region, but not in the epitaxial Si wafer without implantation. On the other hand, no Ni and Cu impurities were observed in the C_3_H_6_-ion-implanted region of any epitaxial Si wafer before the device fabrication process, as shown in Figure 6. These results suggest that C_3_H_6_-ion-implanted defects formed in both single and double epitaxial Si wafers can getter metallic impurities also introduced during the device process.

The amounts of Ni impurity gettered in the C_3_H_6_-ion-implanted region of single and double epitaxial Si wafers, calculated from the SIMS profiles shown in Figure 5b, were 7.3 × 10^11^ and 9.5 × 10^11^ atoms/cm^−2^, respectively. It is interesting to note that the Ni concentration gettered in the implanted region of the C_3_H_6_-ion-implanted double epitaxial Si wafer was higher than that in the C_3_H_6_-ion-implanted single epitaxial Si wafer, in spite of the same C peak concentration in the C_3_H_6_-ion-implanted regions of both samples. Additionally, Figure 5c shows that only the C_3_H_6_-ion-implanted double epitaxial Si wafer slightly gettered Cu. These results indicate that the C_3_H_6_-ion-implanted region of the double epitaxial Si wafer has a high gettering capability for metallic impurities introduced during the device process.

Russo et al. demonstrated that Ni and Cu contamination affects the dark current generation in a CMOS image sensor [28]. The Cu contamination in particular has the greater effect. Hence, it is considered that the higher gettering capability for Ni and Cu contamination in the C_3_H_6_-ion-implanted region of the double epitaxial Si wafer greatly contributed to the suppression of the impurity-related defect formation in the active region of devices, resulting in the reduction in white spot defect number. Thus, the specific DCS signal peaks (Peak 1 and 3) shown in Figure 3 may have originated from the metallic-impurity-related defects, such as Ni and Cu.

**Figure 7:**
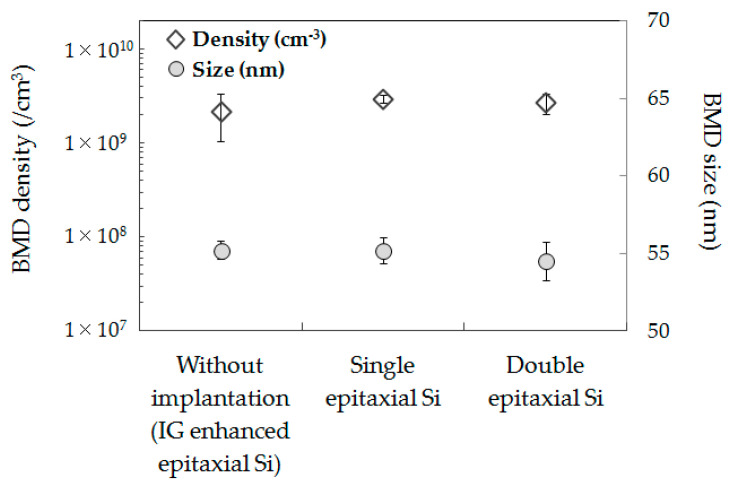
BMD densities and sizes determined by optical microscopy observation on the epitaxial Si wafer without implantation (intrinsic gettering (IG)-enhanced epitaxial Si wafer) and C_3_H_6_-ion-implanted single and double epitaxial Si wafers after the device fabrication process.

Next, the BMD formation behavior in all wafers formed on the Si bulk was investigated. Figure 7 shows BMD densities and sizes in all epitaxial Si wafers after the device fabrication processes.

The CMOS image sensor fabricated in this study used IG-enhanced CZ-Si substrates for all wafers. As shown in Figure 7, no clear difference in BMD density or size was observed for all wafers. It is known that the gettering capability for metallic impurities by BMD is determined by the BMD density and size [31,32]. The gettering capability for metallic impurities on BMD is expected to be the same between C_3_H_6_-ion-implanted single and double epitaxial Si wafers, and it is probably the same also for epitaxial Si wafers without implantation. Therefore, these findings suggest that the gettering capability in the C_3_H_6_-ion-implanted region dominates the reduction in the white spot defects of CMOS image sensor pixels in the double epitaxial Si wafer, rather than the IG by BMD.

### 3.2. Diffusion Behavior of O and H on C_3_H_6_-Ion-Implanted Double Epitaxial Si Wafers

**Figure 8:**
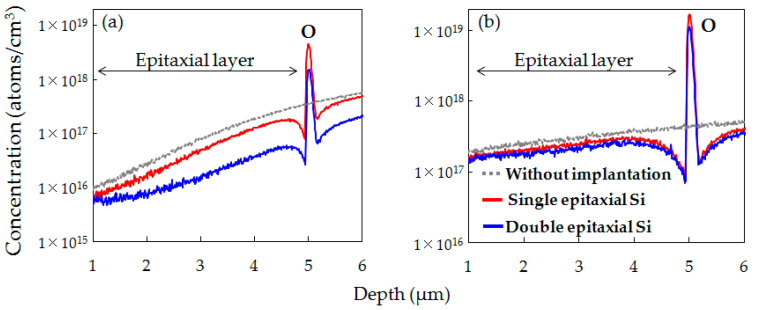
SIMS depth profiles of O concentration in epitaxial Si wafer without implantation and C_3_H_6_-ion-implanted single and double epitaxial Si wafers (**a**) before and (**b**) after device fabrication.

Figure 8a,b respectively shows the SIMS depth profiles of O concentrations before and after the device fabrication processes for the epitaxial Si wafer without implantation and the C_3_H_6_-ion-implanted single and double epitaxial Si wafers. In the C_3_H_6_-ion-implanted single and double epitaxial Si wafers, O was gettered in the C_3_H_6_-ion-implanted region both before and after the device fabrication process. The O peak concentration after the device fabrication process in both samples increased compared to before the device fabrication process. This suggests that the C_3_H_6_-ion-implanted region in not only single but also double epitaxial Si wafers acts as a diffusion barrier preventing O out-diffusion from the CZ-Si substrate. On the other hand, it was found that the O peak concentration of the C_3_H_6_-ion-implanted double epitaxial Si wafer after the device fabrication process was lower than that of the single epitaxial Si wafer. It seems that the lower the O concentration in the C_3_H_6_-ion-implanted region, the higher gettering capability for Ni and Cu.

Additionally, SIMS profiles in Figure 8b indicate that the O concentration in the epitaxial layer of the C_3_H_6_-ion-implanted double epitaxial Si wafer was the lowest, probably owing to the low O concentration in the C_3_H_6_-ion-implanted layer and the effects of the diffusion barrier preventing O out-diffusion. O impurities in the active region of devices cause dark current and contribute to generating white spot defects via the formation of oxygen-related deep levels [17,18,19]. Therefore, it is considered that the low O concentration in the epitaxial layer, which is the active region of devices, also contributes to the reduction in white spot defects of the C_3_H_6_-ion-implanted double epitaxial Si wafer.

**Figure 9:**
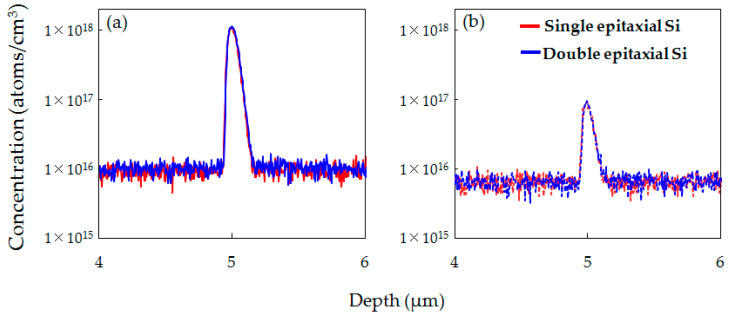
SIMS depth profiles of H concentration of C_3_H_6_-ion-implanted single and double epitaxial Si wafers (**a**) before and (**b**) after device fabrication.

Figure 9 shows the SIMS depth profiles of H concentrations before and after the device fabrication processes of the C_3_H_6_-ion-implanted single and double epitaxial Si wafers. SIMS profiles show that H was gettered in the C_3_H_6_-ion-implanted region of single and double epitaxial wafers, and its peak concentration both before and after the device fabrication process was the same in the single epitaxial Si wafer and double epitaxial Si wafer. Additionally, the peak concentration of H gettered in the C_3_H_6_-ion-implanted region of both wafers was decreased after device fabrication. This decrease in H peak concentration in the C_3_H_6_-ion-implanted region indicates H diffusion to the epitaxial layer and Si bulk during the device fabrication process.

Okuyama et al. reported that H diffused from a hydrocarbon-molecular-ion-implanted region can reduce interface state defects at the Si/SiO_2_ interface, such as P_b_ centers (dangling Si bonds) [33]. Additionally, Yamaguchi et al. demonstrated that a hydrocarbon-moecular-ion-implanted Si wafer can reduce dark current through the passivation of interface state defects at the Si/SiO_2_ interface by H diffused from the hydrocarbon-molecular-ion-implanted region during the device fabrication process [34]. The amounts of H in single and double epitaxial Si wafers out-diffused during the device fabrication process are 8.1 × 10^12^ and 8.5 × 10^12^ cm^−2^, respectively, calculated from the difference in integral values in a 1.0 μm region around the H peak concentration determined by SIMS analysis before and after device processes. It is known that the Si(100)/SiO_2_ interface state defect density in metal-oxide-semiconductor (MOS) transistors is approximately from 10^10^ to 10^11^ cm^−2^ [35]. This indicates that the amount of H diffused from the implanted region of single and double epitaxial Si wafers during the device fabrication process is higher than the interface state density, and this amount is sufficient to passivate the interface state defects at the Si/SiO_2_ interface.

The interface state defects at the Si/SiO_2_ interface increase the mean dark current of the pixels [12]. The results of device performance shown in Figure 4b demonstrated that the dark current of both types of C_3_H_6_-ion-implanted wafers was lower than that of epitaxial wafer without implantation. Thus, the reduction in the dark current of both types of C_3_H_6_-ion-implanted wafers relative to that of the epitaxial wafer without implantation may be due to the reduction in the number of interface state defects by H passivation. On the other hand, a marked reduction in white spot defects, shown in Figure 4a, of the C_3_H_6_-ion-implanted double epitaxial wafer was observed compared with that of the single epitaxial wafer, although the amount of out-diffused H on both wafers was the same. We therefore assumed that the C_3_H_6_-ion-implanted double epitaxial wafer can be expected to have the same effect on the reduction in interface state defects by H passivation as the conventional single epitaxial Si wafer, resulting in the reduction in dark current. The reduction in the number of interface state defects, however, does not directly contribute to marked reduction in the white spot defects of the C_3_H_6_-ion-implanted double epitaxial wafer.

### 3.3. Gettering Sinks of C_3_H_6_-Ion-Implanted Double Epitaxial Si Wafers

In Section 3.1, we confirmed that the higher gettering capability for metallic impurities in the C_3_H_6_-ion-implanted region of double epitaxial Si wafer is the dominant factor behind the considerable reduction in white spot defects of CMOS image sensor pixels. To clarify the increase in the gettering capability for metallic impurities of the C_3_H_6_-ion-implanted double epitaxial Si wafer, the structure, morphology, and electrical property of defects formed in the C_3_H_6_-ion-implanted region of the double epitaxial Si wafer were analyzed by TEM, L-APT, and RTPL, respectively.

**Figure 10:**
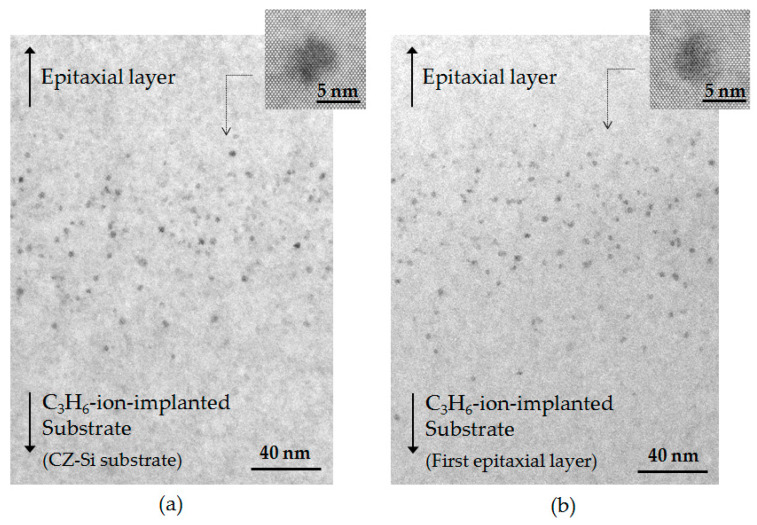
Cross-sectional TEM images of C_3_H_6_-ion-implanted region of (**a**) single and (**b**) double epitaxial Si wafers after device fabrication.

Figure 10a,b respectively shows TEM cross-sectional images of the C_3_H_6_-ion-implanted region of the single epitaxial wafer and double epitaxial wafer after the device fabrication process. The TEM images in the C_3_H_6_-ion-implanted region of both wafers only showed defects 5 nm in size. These defects were also observed in a monomer-ion-implanted Si wafer and hydrocarbon-molecular-ion-implanted Si wafer after heat treatment [36,37]. It has been reported that the 5 nm defects are agglomerates consisting of C and *I* (C–*I* agglomerates), and those defects act as gettering sinks for metallic impurities [37]. It is considered that Cu and Ni gettering in the C_3_H_6_-ion-implanted region of both single and double epitaxial Si wafers shown in Figure 5 occurred through this type of defect. However, the 5 nm defect densities calculated by counting from three different TEM images of C_3_H_6_-ion-implanted single and double epitaxial Si wafers are 9.0 × 10^16^ and 8.7 × 10^16^ cm^−2^, respectively, which were not significantly different. Additionally, no extended defects, such as dislocation, that lead to an increase in metallic impurity gettering were observed in the C_3_H_6_-ion-implanted region of the double epitaxial Si wafer after the device fabrication process.

In a previous study, we demonstrated that these 5 nm defects induced by a high C_3_H_6_-ion implantation dose of 1 × 10^16^ cm^−2^ in the CZ-Si substrate and the first epitaxial layer also consisted of C and *I* agglomerates [38]. L-APT data in high-dose C_3_H_6_-ion-implanted samples indicated a difference in the O distribution between the 5 nm defects induced in the CZ-Si substrate and the first epitaxial layer [38,39]. This implies a significant difference in the morphology of 5 nm defects between the CZ-Si substrate and the first epitaxial layer. Hence, an understanding of the O distribution at the atomic level around these defects after the device fabrication process is expected to be the key to reduce the white spot defects of the CMOS image sensor in the C_3_H_6_-ion-implanted double epitaxial Si wafer.

**Figure 11:**
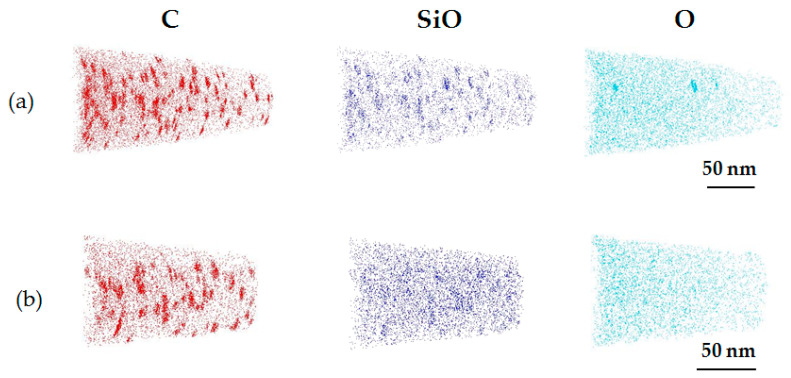
3D L-APT maps of C- and O-related components (SiO and O) of the C_3_H_6_-ion-implanted region in C_3_H_6_-ion-implanted (**a**) single and (**b**) double epitaxial Si wafers.

Figure 11 shows the three-dimensional (3D) distribution of C- and O-related components (SiO and O) of the C_3_H_6_-ion-implanted region in single and double epitaxial Si wafers measured by L-APT. C agglomerates can be observed in the L-APT map of C in both wafers. It is considered that the C agglomerates correspond to defects observed by TEM shown in Figure 10 in both samples, that is, agglomerates consisting of C and *I* (C–*I* agglomerates) [38]. Additionally, L-APT maps show that O-related components also formed agglomerates in both wafers. The formation areas of O agglomerates corresponded to the formation areas of C agglomerates, which is particularly marked in the C_3_H_6_-ion-implanted single epitaxial Si wafer. In C_3_H_6_-ion-implanted double epitaxial Si wafer, it is seen that many O-related components were uniformly distributed without the formation of O agglomerates. It is considered that the difference in the O distribution between the C_3_H_6_-ion-implanted single and double epitaxial Si wafers comes from the difference in the O concentration in the C_3_H_6_-ion-implanted region, shown in Figure 8.

**Figure 12:**
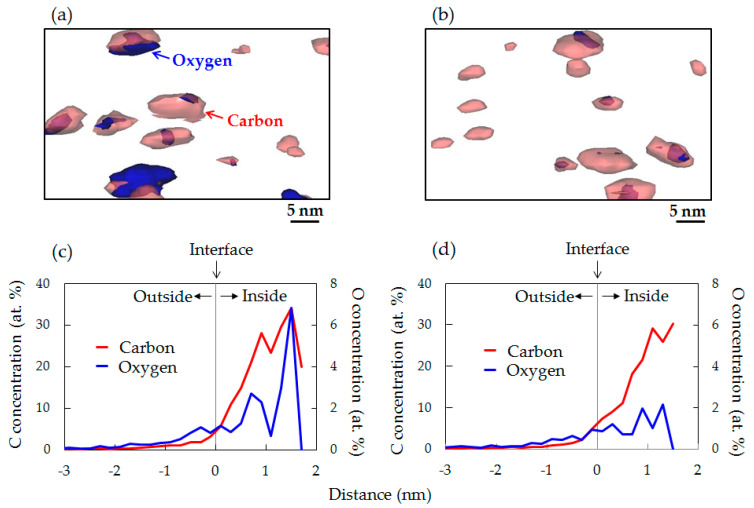
Isoconcentration surface of 3 at.% C and 1.8 at.% O in C_3_H_6_-ion-implanted (**a**) single and (**b**) double epitaxial Si wafers, and proxigrams for C and O at the C isoconcentration interface on C_3_H_6_-ion-implanted (**c**) single and (**d**) double epitaxial Si wafers. The volume of extracted maps is 50 × 50 × 30 nm^3^.

The isoconcentration surfaces of ≥3 at.% C (red) and ≥1.8 at.% O (blue) extracted from the interior of acicular samples around the center are shown in Figure 12a,b, to clearly illustrate the distribution of O around the C agglomerates. The O drawn on the isoconcentration surface includes the concentration of O in the SiO component. The isoconcentration surfaces of C drawn as spheres delineate border regions of 3 at.% C. Inside the spheres, C exceeds 3 at.%. The isoconcentration surface of C, therefore, may be similar to that of C agglomerates (C–*I* agglomerates). The isoconcentration surface maps in both wafers indicate that the isoconcentration surface of O was distributed almost at the same position as the isoconcentration surface of C. The results of isoconcentration surface maps shown in Figure 12a,b demonstrate that C–*I* agglomerates are gettering sinks for O diffused during the device process. Additionally, in Figure 12a,b we can confirm the spheres were composed of only the isoconcentration surface of C. It is considered that agglomerates with only the isoconcentration surface of C are C–*I* agglomerates that include few or no O atoms.

The proximity histograms (proxigrams) were calculated by IVAS to reveal the evolution of the C and O concentrations from the isoconcentration surfaces of C. Figure 12c,d shows proxigrams for C and O of C_3_H_6_-ion-implanted single and double epitaxial Si wafers, respectively. These results are average values calculated from the isoconcentration surface of C excluding the isoconcentration surface at the edge of the acicular samples. The C concentration increased toward the inside of isoconcentration surfaces of C in both samples. This indicates that the C concentration of agglomerates is higher toward the center. In the C_3_H_6_-ion-implanted single epitaxial Si wafer shown in Figure 12c, although O also exists outside the isoconcentration surface of C, most of the O is distributed inside C agglomerates that have high C concentrations along the distribution of C. In comparison, in the C_3_H_6_-ion-implanted double epitaxial Si wafer shown in Figure 12d, O is distributed inside C agglomerates, but its concentration is much lower than that in the single epitaxial Si wafer. These observations suggest that the concentrations of O in the defects (C–*I* agglomerates) differ greatly between single and double epitaxial Si wafers, although they are regarded as the same type of defect in the TEM observation.

In the previous study, we found that defects formed upon C_3_H_6_-ion-implantation at a high dose of 1 × 10^16^ cm^−2^ in the first epitaxial layer are C–*I* agglomerates without O atoms [38,39]. This tendency of the O distribution in defects in the previous study is in good agreement with that of defects in the double epitaxial Si wafer formed after the device process in the current study. Hence, the distribution of O in the defects formed upon C_3_H_6_-ion-implantation in the epitaxial layer is the same regardless of implantation dose and annealing condition. This finding suggests that the low O concentration in the defects is a factor behind the higher gettering capability for metallic impurities. The formation of this type of defect probably can be achieved by C_3_H_6_-ion-implantation into the low-O-concentration layer.

**Figure 13:**
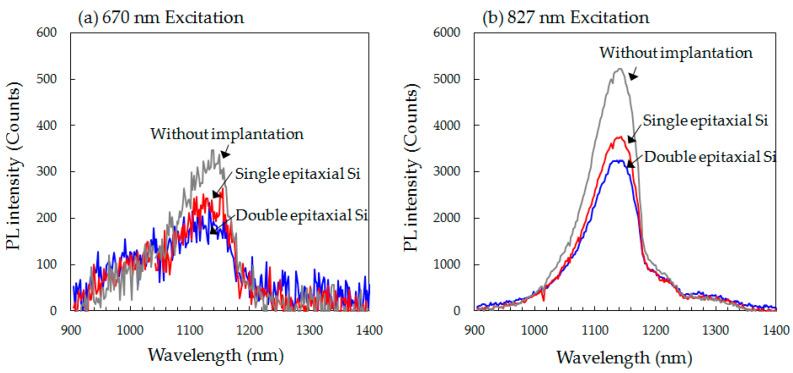
RTPL spectra under (**a**) 670 nm and (**b**) 827 nm excitation in the epitaxial Si wafer without implantation and C_3_H_6_-ion-implanted single and double epitaxial Si wafers before CMOS device fabrication.

In addition to the defect morphology, we consider the impact of O atoms included in the defects (C–*I* agglomerates) on the electrical properties of the defects by RTPL measurement on all wafers before device fabrication. Figure 13 shows the RTPL spectra under 670 and 827 nm excitations in the epitaxial Si wafer without implantation and C_3_H_6_-ion-implanted single and double epitaxial Si wafers.

We found that the RTPL intensity of both epitaxial Si wafers implanted with C_3_H_6_ ions markedly decreased compared with that in the case without implantation. In addition, the RTPL intensity of the double epitaxial Si wafer at both excitation levels was lower than that of the single epitaxial Si wafer. Yoo and coworkers [40,41] reported that the RTPL intensity due to band-to-band emission is very sensitive to the number of implantation defects that are electrically activated in the Si wafer. C_3_H_6_-ion-implantation defects were formed at a depth of approximately 5 μm from the wafer surface, in this study. At the penetration depths of 670 and 827 nm excitations (around 4 and 10 μm), most of the excited carriers contributing to the PL emission were found around the implanted region. Excited carriers that diffused into the implanted region recombined at the nonradiative defects therein, resulting in an RTPL intensity decrease. We therefore infer that the decrease in the RTPL intensity of C_3_H_6_-ion-implanted wafers was caused by recombination at C_3_H_6_-ion-implantation defects, and that the number of such defects is higher in the double epitaxial Si wafer than in the single epitaxial wafer. It is considered that the difference in the number of defects between the single and double epitaxial Si wafers observed by RTPL corresponds to the difference in the number of C–*I* agglomerates. This also indicates the difference in the number of gettering sinks that exist before the device fabrication process.

We consider that gettering of metallic impurities on the C_3_H_6_-ion-implanted double epitaxial Si wafer occurs owing to the segregation mechanism involving C–*I* agglomerates, as no extended defects were observed in C_3_H_6_-ion-implanted region, as shown in Figure 10. The segregation gettering mechanism is known for Fe gettering in p/p^+^ wafers; this gettering capability depends on the B concentration in the p^+^-Si substrate [42,43]. It is believed that the high binding energy of Fe and B enhances the solid solubility of Fe in the p^+^-Si substrate. Jin and Dunham reported that defects consisting of C and *I*, such as C_3_–*I*_3_, have high binding energies with metallic impurities such as Cu and Ni [44]. Hence, it is considered that C–*I* agglomerates formed upon C_3_H_6_-ion implantation can be effective gettering sinks for metallic impurities because of their strong interaction with metallic impurities. If gettering of metallic impurities in the C_3_H_6_-ion-implanted region occurs by the segregation mechanism, the gettering capability should be dependent on the number of C–*I* agglomerates. However, the number of C–*I* agglomerates observed by TEM did not differ between single and double epitaxial wafers.

A recent L-APT study on Cu gettering in the hydrocarbon-molecular-ion-implanted region revealed that the Cu gettering capability through C–*I* agglomerates decreased with increasing number of O atoms in the agglomerates [45]. Our L-APT data in Figure 11 show that the O concentration included in C–*I* agglomerates was much lower in the C_3_H_6_-ion-implanted double epitaxial Si wafer than in the single epitaxial Si wafer, even after the device fabrication process. It is considered that the O concentration in the defects of the double epitaxial Si wafer was even lower before the device fabrication process, as there was no diffusion of O from the substrate. Additionally, according to the previous report of Medernach and coworkers, O atoms contribute partially to the passivation of electrically activated point defect clusters, resulting in the reduction in gettering capability for metallic impurities [46]. RTPL results in Figure 12 also showed that the number of defects formed in the C_3_H_6_-ion-implanted region was higher for the double epitaxial Si wafer than for the single epitaxial Si wafer. Therefore, we assume that O atoms included in C–*I* agglomerates acted to reduce the electronic properties of agglomerates, which means a decrease in the number of gettering sinks for metallic impurities. The gettering of metallic impurities in the C_3_H_6_-ion-implanted region occurs because of the high binding energy between metallic impurities and C–*I* agglomerates and also depends on the O concentration in C–*I* agglomerates.

**Figure 14:**
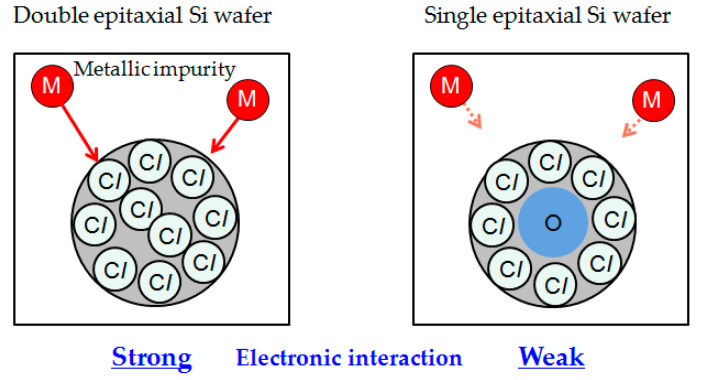
Illustration of model of gettering reaction of C_3_H_6_-ion-implanted Si wafers. Modified from Onaka-Masada et al. [38], Copyright (2018) IEEE.

Hence, it is inferred that the existence of C–*I* agglomerates that have low O concentrations is responsible for the high gettering capability of the C_3_H_6_-ion-implanted double epitaxial Si wafer and, thus, directly affects the white spot defect number of CMOS image sensor pixels. The gettering reaction for metallic impurities introduced during the device fabrication process can be explained by the same mechanism as that of the gettering reaction for intentionally contaminated metallic impurities described in Onaka-Masada et al. [38]. The mechanism behind the gettering reaction on the C_3_H_6_-ion-implanted double epitaxial wafer is illustrated in Figure 14.

### 3.4. Appropriate Design of Gettering Layer for 3D-Stacked BSI CMOS Image Sensors

In the fabrication of 3D-stacked BSI CMOS image sensors, a thin-wafer fabrication process is needed for the formation of the light-receiving surface of the photodiode. The device Si wafer is reduced to a thickness of less than 10 μm by mechanical grinding and CMP and then to the final target thickness by other etching processes [47]. Consequently, gettering sinks formed in the Si substrate of the device Si wafer, such as the IG substrate, are eliminated by the mechanical and chemical processes used in thin-wafer fabrication. However, specifically, the introduction of metallic impurities such as Cu during stacked BSI image sensor fabrication mainly occurs in the thin-wafer fabrication process and the Cu damascene electrodeposition process, owing to mechanical grinding and CMP. In particular, Cu atoms originating from Cu TSV diffuse into the device active region of the photodiode even with a low-temperature thermal budget [48,49]. Therefore, it is considered that the formation of a gettering layer that remains until the final stage of the thin-wafer formation process is indispensable for the fabrication of high-performance 3D-stacked BSI CMOS image sensors that also have a high gettering capability for metallic impurities.

Additionally, the passivation effect of interface states upon H sintering treatment in the BSI process may be less than that in the FSI process [50,51,52]. Vici et al. also reported that interface state defects are created by BSI processes such as wafer bonding and thinning [53]. The 3D-stacked CMOS image sensor fabrication includes many bonding and thinning processes. The increase in the density of interface state defects at the Si/SiO_2_ interface in the isolation region or transistor gate oxide leads to increasing dark current and RTS noise.

The double epitaxial Si wafers in which the first epitaxial layer is used as an etch stop layer can be selected as a starting material for the device Si wafer of stacked BSI CMOS image sensors. If a double epitaxial Si wafer implanted with hydrocarbon molecular ions in the epitaxial etch stop layer is used as a starting material for 3D-stacked BSI image sensors, the gettering layer can remain until the final stage of the thin-wafer process compared with the epitaxial wafer using an IG substrate and even a hydrocarbon-molecular-ion-implanted CZ-Si substrate. The results of device performance shown in Figure 3 and Figure 4a demonstrated that the C_3_H_6_-ion-implanted double epitaxial Si wafer can drastically decrease the white spot defects of CMOS image sensor pixels comparing with single epitaxial Si wafer. In addition, C_3_H_6_-ion-implanted double epitaxial Si wafer is expected to have a reduced number of interface state defects, owing to H termination, because the concentration of H diffused from the C_3_H_6_-ion-implanted region during device fabrication was over ten times higher than the density of interface states. The use of the hydrocarbon-molecular-ion-implanted double epitaxial wafer means that gettering sinks for H also remain until the final stage of fabrication. Okuyama et al. demonstrated that the Si wafer implanted with hydrocarbon molecular ions had a reduced number of interface state defects at the Si/SiO_2_ interface, even in the temperature range in which the dissociation of H on the passivated defects occurs [33]. This is because H continues to be provided to the Si/SiO_2_ interface from the hydrocarbon-molecular-ion-implanted region during heat treatment. Thus, hydrocarbon-molecular-ion-implanted defects in the double epitaxial wafer can be effective storage tanks for H during 3D-stacked BSI fabrication. The use of the hydrocarbon-molecular-ion-implanted double epitaxial wafer is expected to decrease the number of interface state defects during the fabrication of stacked BSI CMOS image sensors.

We believe that the Si wafer with a gettering layer formed by hydrocarbon-molecular-ion implantation into the epitaxial layer can be suitable for 3D-stacked BSI image sensors and will realize high-performance CMOS image sensor fabrication.

## 4. Conclusions

We investigated the dark current characteristics of CMOS image sensor pixels with C_3_H_6_-ion-implanted double epitaxial Si wafers. The following is concluded.

(1) Considerable reduction in white spot defects by using a C_3_H_6_-ion-implanted double epitaxial Si wafer was demonstrated in the fabrication of CMOS image devices.
(2) The reduction in white spot defects of the C_3_H_6_-ion-implanted double epitaxial Si wafer occurred owing to the high gettering capability for metallic impurities introduced during the device fabrication process and the suppression of O diffusion into the device active layer. The higher gettering capability of the C_3_H_6_-ion-implanted double epitaxial Si wafer was also effective for metallic impurities introduced during device fabrication.
(3) H at a concentration of 8.5 × 10^12^ cm^−2^ was confirmed to diffuse from the C_3_H_6_-ion-implanted region of the double epitaxial Si wafer during device fabrication. This H concentration was over ten times higher than the density of interface states. The C_3_H_6_-ion-implanted double epitaxial wafer has the same effect on the reduction in dark current as C_3_H_6_-ion-implanted single epitaxial Si wafer; however, H gettered in the C_3_H_6_-ion-implanted region does not contribute to the marked reduction in white spot defects compared with that in the C_3_H_6_-ion-implanted single epitaxial Si wafer.
(4) L-APT results indicated that the O concentration in defects was different between the C_3_H_6_-ion-implanted single and double epitaxial Si wafers. The O concentration in defects determines the number of gettering sinks for metallic impurities. The defects that have low O concentrations formed in the C_3_H_6_-ion-implanted double epitaxial Si wafer locally enhance the solid solubility for metallic impurities, owing to the strong interaction between the defects and metallic impurities.

We believe that our C_3_H_6_-ion-implanted double epitaxial Si wafers can contribute to improving the performance of 3D-stacked BSI image sensors.
